# Do Randomized Controlled Trials Discuss Healthcare Costs?

**DOI:** 10.1371/journal.pone.0012318

**Published:** 2010-08-23

**Authors:** G. Michael Allan, Christina Korownyk, Kate LaSalle, Ben Vandermeer, Victoria Ma, Douglas Klein, Donna Manca

**Affiliations:** 1 Department of Family Medicine, University of Alberta, Edmonton, Alberta, Canada; 2 Institute of Health Economics, Edmonton, Alberta, Canada; 3 Towards Optimized Practice, Edmonton, Alberta, Canada; 4 Alberta Research Centre for Health Evidence, University of Alberta, Edmonton, Alberta, Canada; 5 Continued Professional Learning, University of Alberta, Edmonton, Alberta, Canada; 6 Alberta Family Practice Research Network, Edmonton, Alberta, Canada; Yale University School of Medicine, United States of America

## Abstract

**Background:**

Healthcare costs, particularly pharmaceutical costs, are a dominant issue for most healthcare organizations, but it is unclear if randomized controlled trials (RCTs) routinely discuss costs. Our objective was to assess the frequency and factors associated with the inclusion of costs in RCTs.

**Methods and Findings:**

We randomly sampled 188 RCTs spanning three years (2003-2005) from six high impact journals. The sample size for RCTs was based on a calculation to estimate the inclusion of actual drug costs with a precision of +/−3%. Two reviewers independently extracted cost data and study characteristics. Frequencies were calculated and potential characteristics associated with the inclusion of costs were explored. Actual drug costs were included in 4.7% (9/188) of RCTs; any actual costs were included in 7.4% (14/188) of RCTs; and any mention of costs was included in 27.7% (52/188) of RCTs. As the amount of industry funding increased across RCTs, from non-profit to mixed to fully industry funded RCTs, there was a statistically significant reduction in the number of RCTs with any actual costs (Cochran-Armitage test, p = 0.005) and any mention of costs (Cochran-Armitage test, p = 0.02). Logistic regression analysis also indicated funding was associated with the inclusion of any actual cost (OR = 0.34, p = 0.009) or any mention of costs (OR = 0.63, p = 0.02). Journal, study conclusions, study location, primary author's country and product age were not associated with inclusion of cost information.

**Conclusion:**

While physicians are encouraged to consider costs when prescribing drugs for their patients, actual drug costs were provided in only 5% of RCTs and were not mentioned at all in 72% of RCTs. Industry funded trials were less likely to include cost information. No other factors were associated with the inclusion of cost information.

## Introduction

The costs of pharmaceuticals in almost all countries of the Organisation for Economic Co-operation and Development (OECD) are increasing faster than any other aspect of healthcare budgets [1]. Many countries struggle with a wide variety of strategies to reduce or control pharmaceutical spending [2], [3], but many approaches increase drug costs for patients, often worsen outcomes and shift costs to other areas [4]–[8]. Surveys have found that physicians consider health care costs to be important when prescribing, even when patients have full drug coverage [9]–[11]. Other studies have observed that physicians' prescribing was responsive to price when costs were made available to them [12]–[14]. A number of studies in the clinical setting have found that prescribing costs declined without negative impacts on care if physicians were given feedback about their prescribing costs and provided with cost information [15]–[17]. Ideally, physicians would select less expensive products if the alternatives were equally efficacious. Unfortunately, physicians have a very limited understanding of drug cost and, more importantly, do not understand the large cost differential between expensive and inexpensive drugs [18].

Physicians have indicated they want more cost information [18], although several researchers have reported that cost information was difficult to obtain [19], [20]. Currently, there are no data quantifying the availability of this information in the medical literature.

Our objective was to determine how often actual drug costs and healthcare costs in general were included in randomized controlled trials (RCTs) and to identify the factors that may be associated with the inclusion of cost information in RCTs.

## Methods

Although there are no established templates for studies assessing reporting in the literature, PRISMA [21], the guide for reporting of systematic reviews, serves as a reasonable model and was followed where possible.

### Eligibility Criteria

Our study was limited to RCTs, the gold-standard of therapy evidence. To address the inclusion of drug costs, only RCTs comparing a pharmaceutical to no treatment, placebo or active control were eligible.

### Information Sources

Similar to past studies that assessed reporting in the literature [22], [23], we used six major general medical journals to sample RCTs: New England Journal of Medicine (N Eng J Med), Journal of the American Medical Association (JAMA), Lancet, British Medical Journal (BMJ), Annals of Internal Medicine (Ann Intern Med) and Archives of Internal Medicine (Arch Intern Med).

### Search

We used PubMed to identify all RCTs. A search was performed in May 2006 using the abbreviated journal title restricted to the date range January 1 to December 31 for each individual year (2003, 2004 or 2005) and Randomized Controlled Trial for type of article. For example, to search for RCTs published in the N Engl J Med in 2005, we entered N Engl J Med in the search bar and limited the search to RCTs under type of article and the dates to January 1, 2005 to December 31, 2005. To confirm these searches captured all the RCTs published in journals, we reviewed each issue from two of journals for 2005 and found none were missed. The PubMed search did capture a few articles that were not RCTs but these were identified during review for inclusion.

### Study Selection

The total number of articles found in each search of article type, journal and year (e.g., RCTs in N Engl J Med in 2005) was used to generate the randomization for that group. Randomization was performed using Excel.

The sample size was based on our objective to obtain an accurate and precise estimate of reporting of actual drug costs in RCTs. Initially, 10 articles were randomly selected from each of the six journals published in the year 2005. An interim analysis of those 60 RCTs found that actual drug costs were reported in less than 5% of the RCTs. To attain a precision of +/− 3% with a 95% Confidence Interval (CI), 188 RCTs had to be reviewed, assuming the reporting of actual cost was ≤5%. In addition to 10 RCTs from 2005 from each journal, 10 RCTs were randomly selected from each journal for the years 2003 and 2004. Eight more studies were randomly selected from any of the included years or journals.

The abstract of each article randomly selected was reviewed to confirm it met the eligibility criteria: an RCT that assessed pharmaceutical therapy. If it did not, a random number was again generated to choose a replacement RCT.

### Data Abstraction

To identify cost information, two authors (GMA & KL) independently searched each RCT with the Adobe Reader Search function using the key words “cost”, “pric”, “$”, “dollar”, “pound”, “£”, “money”, “fee”, “fund”, “econom”, “financ”, and “expens”. One author (KL) also read each RCT for any mention or discussion of costs. We classified costs in to three categories: actual drug costs, any actual costs, and any mention of costs. ‘Actual drug costs’ were any specific drug cost provided as a numeric value in dollars, pounds or other currency. ‘Any actual costs’ were any drug cost and/or any healthcare cost provided as a numeric value in dollars, pounds or other currency. ‘Any mention of costs’ was considered any of the above plus any discussion of health care costs, no matter how general (e.g. the management of this condition is very expensive). We limited counting ‘any mention of costs’ to once per paragraph when costs were discussed without numeric values. This was because a single paragraph may have frequently included words such as “cost”, “expense”, or “economic”, but the rest of the article had no other discussion of costs. We did not count costs relating to expenses of the trial (e.g. “we paid participants $5 to complete the testing”), funding sources or financial conflict of interest. Finally, we did not count costs-associated words appearing in reference sections.

Two authors (GMA and CK) independently abstracted data on RCT characteristics: experimental drug, age of the experimental drug (novel or established), the comparator, funding of the trial, study country, primary author's country and conclusion. We defined the age of the experimental drug as “novel” if it was still on-patent at the time of the study and as “established” if it was off-patent at the time of the study. Funding was divided into categories of industry, non-profit or mixed. Funding was classified as mixed if the study had both non-profit and industry funding sources. If a study had any industry funding, even if only supplying the pharmaceutical product, it was classified as mixed. We found that classifying conclusions as simply “positive” or “negative” did not reflect the range of language used by authors to explain their results. Therefore, we modified the classification used by Kjaergard and Als-Nielsen [24] by shortening their ranking of 6 into 4 (combining the ranks of 5 & 4 and 2 & 3). Our four options were: Positive, Positive But, Negative But and Negative. Any disagreements were resolved with discussion and consensus. The data abstraction template is available in Appendix S1. In extracting cost information, we originally had more sub-categories but found the frequency of cost reporting so infrequent it did not warrant multiple sub-categories.

### Data Analysis

Similar to previous studies [25], [26] that assessed reporting in the literature, we compared the data extraction from a sample of the RCTs to determine inter-rater agreement. We used the abstraction data from two journals (63 RCTs) to compare general agreement over cost and RCT characteristics. We also assessed the *Kappa* values for areas with a higher potential for disagreement: the age of the experimental drug (novel or established), funding of the trial and conclusion.

We summarized the results to provide a descriptive analysis including overall mean percent of studies which had actual drug costs, any actual costs and any mention of costs, with 95% CI. We originally intended to compare the inclusion of actual drug costs or any mention of costs across different study characteristics (e.g., funding or product age). However, RCTs with actual drug costs were so few in number that we opted to analyze any actual costs (actual drug costs or any other healthcare cost with numeric value in a dollars, pounds or any currency) and any mention of cost. We used Fisher-Freeman-Halton test for nominal data to compare the number of RCTs that included actual costs and any mention of costs associated with different journals, primary author's country or study location. We used Cochran-Armitage test for ordinal data to compare the number of RCTs that included actual costs and any mention of costs associated with funding and study conclusions. We used Fisher's Exact Test to compare the number of RCTs that included actual costs and any mention of costs associated with product age (novel or established). We also did a logistic regression to compare the dependant variables of actual cost and mention of cost simultaneously with the independent variables of funding, conclusion, primary author's country, and product age.

## Results

A total of 188 RCTs were reviewed (a full list is available in Appendix S2). Disagreement between reviewers was uncommon. From a sample of 63 RCTs (from two journals), agreement in extracting cost information was 94% and agreement in extraction for RCT variables (such as funding, outcomes, etc) was 91%. The *Kappa* values for the age of the experimental drug (novel or established) was 0.87, funding of the trial 0.74, and the conclusion 0.75. All disagreements were resolved with discussion.

Table 1 reports how often costs were mentioned in RCTs. The number of RCTs with actual drug costs in dollars, pounds or other currency was 9 of 188 or 4.7% (95% CI, 1.7%–7.7%). The number of RCTs with any actual cost (of drugs and/or other healthcare in dollars, pounds or other currencies) was 14 of 188 or 7.4% (95% CI, 3.7%–11.1%), varying among the journals from 3% (1/32) to 19% (6/32) although the difference was not statistically significant (Fisher-Freeman-Halton test, p = 0.17). The number of RCTs with any mention of costs was 52 of 188 or 27.7% (95% CI, 20.6%–33.3%), varying among the journals from 23% (7/31) to 31% (10/32), although the difference was not statistically significant (Fisher-Freeman-Halton test, p = 0.97).

**Table 1:** Inclusion of drug costs, health care cost or any mention of cost in Randomized Controlled Trials.

	Number of articles with actual drug cost*	Number of articles with any actual cost*	Number of articles with any mention of costs	Total number of times costs mentioned
JAMA (31)	1 (3%)	2 (6%)	8 (26%)	15
N Engl J Med (32)	0	1 (3%)	10 (31%)	33
Lancet (32)	5 (16%)	6 (19%)	10 (31%)	59
BMJ (30)	2 (7%)	3 (10%)	8 (27%)	20
Ann Intern Med (31)	0	1 (3%)	7 (23%)	23
Arch Intern Med (32)	1 (3%)	1 (3%)	9 (28%)	16
TOTAL (188)	9 (4.7%)	14 (7.4%)	52 (27.7%)	166

*Actual drug cost and any actual cost means there was a cost with a numeric value in dollars, pounds or other currency.

Costs were mentioned 166 times in the RCTs for a mean of 0.9 mentions per RCT. However, the median number of times that cost was mentioned was 0 because 72% of RCTs did not include any costs. Of the RCTs with any mention of costs (52 of 188), there was an average of 3.2 mentions per RCT and a median of 1.5 mentions per RCT. Half (26 of 52) of the RCTs including costs only mentioned them once. Six of the 188 RCTs had 10 or more (maximum 21) mentions of costs per article and account for 90 of the 166 mentions overall. Therefore, 3% of the articles account for 54% of the total number of times that costs were mentioned.

Table 2 provides the frequency of costs mentioned in RCTs grouped by study characteristics. Comparing RCTs based on funding, there was a statistically significant reduction in the number of RCTs including any actual costs (Cochran-Armitage test, p = 0.005) and any mention of costs (Cochran-Armitage test, p = 0.02) from non-profit to mixed to industry funded studies. Compared to RCTs of novel drugs, RCTs of established drugs were significantly more likely to include any actual costs (fisher's exact test, p = 0.02), although were not more likely to include any mention of costs (fisher's exact test, p = 0.51).

**Table 2:** Study characteristics associated with costs being mentioned in Randomized Controlled Trials.

		Total number	Number of Studies with Actual Costs (%)	Statistical Testing	Number of Studies Mentioning Costs (%)	Statistical Testing
Funding	Industry	68	1 (1%)	p = 0.005*	12 (18%)	p = 0.02*
	Mixed	67	5 (7%)		21 (31%)	
	Non-profit	53	8 (15%)		19 (36%)	
Conclusion	Negative	37	5 (14%)	p = 0.16*	13 (35%)	p = 0.49*
	Negative But	14	1 (7%)		3 (21%)	
	Positive But	34	2 (6%)		8 (24%)	
	Positive	103	6 (6%)		28 (27%)	
Primary Author's Country	United States	86	4 (5%)	p = 0.12†	25 (29%)	p = 0.52†
	Europe	72	5 (7%)		17 (24%)	
	Other	30	5 (17%)		10 (33%)	
Study Location	Multi-centered	53	3 (6%)	p = 0.59†	11 (21%)	p = 0.61†
	United States	57	3 (5%)		16 (28%)	
	Europe	39	3 (8%)		12 (31%)	
	Developing Countries	21	3 (14%)		8 (38%)	
	Other	18	2 (11%)		5 (28%)	
Product Age	Novel	109	4 (4%)	p = 0.02‡	28 (26%)	p = 0.51‡
	Established	79	10 (13%)		24 (30%)	

*Cochran-Armitage test.

† Fisher-Freeman-Halton test.

‡ Fisher's exact test.

Logistic regression analysis indicated that funding was the only factor associated with the inclusion of any actual cost (OR = 0.34, p = 0.009) or any mention of costs (OR = 0.63, p = 0.02). None of the other independent variables (conclusion, product age or primary author's country) were significantly associated with the inclusion of any actual cost or any mention of costs. The significant result of product age associated with the inclusion of any actual cost disappeared in regression analysis, suggesting that the inclusion of any actual costs with product age may be attributed to the correlation between product age and funding (i.e., industry funded RCTs would more likely to study novel drugs still on patent). The correlation coefficient between these two variables was high at 0.54, while no other two variables in the regression analysis had a correlation higher than 0.13.

## Discussion

Healthcare costs were mentioned in only 28% of RCTs of pharmaceutical therapy and actual drug costs were rarely (5%) included. The User's Guide to the Medical Literature [27] suggests that costs be considered in the application of all study results. Furthermore, most physicians believe that costs should be considered in clinical decision-making [9]–[11]. Unfortunately, physicians do not know the costs of drugs [18] or other healthcare interventions [28]. Therefore, if clinicians were to consider costs in evaluating application of an RCT result, the drug cost would have to be included in the paper. It might be argued that physicians could access the costs elsewhere, although some authors have reported accurate costs are difficult to find [19], [20]. The inclusion of costs, even a brief mention, would not be onerous and would help physicians consider and compare the relative benefit of a drug in relation to its' cost.

The total number of times costs were mentioned (166 times) appears to be relatively high, but this number is inflated by a few studies with cost as their primary focus. For example, a Lancet article includes costs 20 times which is equal to or more than all the articles from three of the other journals. We felt that these differences did not warrant statistical testing among the journals as the number of RCTs with any mention of cost did not differ among journals. Additionally, any differences between the total number of mentions could be due to chance since only 3% (6/188) of the RCTs provided 54% (90/166) of the total number of times costs were mentioned. Cost was a focus of these articles in contrast to the other 97% (182/188) of the RCTs.

Inclusion of healthcare costs did not vary by journal, primary author's country, study location, product age or conclusion. Although the inclusion of any actual cost was statistically less common in established (or older) drugs compared to novel (or newer), this finding disappeared on logistic regression. The association to product age and the inclusion of any actual cost was due to the correlation of funding and product age. Funding appears to be the only factor associated with the inclusion of cost information, as identified by the inclusion of any actual cost or any mention of cost in bi-variant analysis and multi-variant analysis of logistic regression. Industry funded studies were significantly less likely to mention costs than studies funded solely from non-profit sources. In fact, the likelihood that cost information was included in RCTs increased as the degree of industry funding declined. RCTs with any mention of costs increased across funding groups: from industry (18%) to mixed (31%) to non-profit (36%). This pattern was consistent with the inclusion of any actual costs: from industry (1%) to mixed (7%) to non-profit (15%) funding.

In light of previous studies showing that funding was associated with study conclusion [22]–[24], [29], [30], it may be reasonable to conclude that funding could also be associated with the writing of the articles. It is possible that authors of industry-funded trials avoid discussion of costs because their products are usually more expensive. However, it is also possible that the authors of non-profit funded trials are more likely to include a discussion of costs because they feel obliged to consider costs as many non-profit sources (like government-linked sponsors) may be more cost-conscious.

A potential limitation of the study is the four-year delay from search to submission as cost reporting may have changed in that time. This is, however, highly unlikely as no incentive or initiative has been introduced to trigger such as change. Our journal selection may have impacted our results, particularly compared to journals with a health economic focus. Additionally, we included only RCTs — other types of articles may have included more cost information. That said, our goal was to examine clinically relevant RCTs in high impact clinical journals that are more often read and used by clinicians. As data extraction required some interpretation, particularly for the subjective areas such as study conclusion, there is the potential that different raters would categorize the conclusions differently. Our level of agreement was similar to investigators in previous studies assessing reporting in the literature. Our *Kappa* ranged from 0.87 to 0.74 while Pitkin *et al* [25] reported a 0.81 *Kappa* and Moher *et al* [26] report a *Kappa* values between 1 and 0.53.

Future research should examine other medical information resources, such as internet evidence-based summarized sites, other types of articles and perhaps other journals. If other resources provide cost information, the accuracy of the information should be examined, although defining true cost and accounting for cost difference over time and different locations will be challenging.

In summary, actual drug costs or any actual costs are rarely included in RCTs and costs are not mentioned at all in 72% of RCTs. Trials funded from non-profit sources were more likely to include cost information compared to trials with solely industry based funding. No other factors were associated with cost inclusion. More work is needed to provide physicians with the cost information that can inform their decision-making and potentially reduce healthcare costs.

## Supporting Information

Appendix S1 Data Abstraction Form
(0.06 MB DOC)

Appendix S2 Articles Included
(0.11 MB DOC)
