# Supplementary material for: Do Randomized Controlled Trials Discuss Healthcare Costs?
Source: PLoS One. 2010 Aug 23;5(8):e12318. doi: 10.1371/journal.pone.0012318 (PMC2925897; doi:10.1371/journal.pone.0012318)
Supplement: Appendix S1 — Data Abstraction Form (0.06 MB DOC) [file pone.0012318.s001.doc]

**Appendix S1: Data Abstraction Form**

| Article Number | | |  |
| --- | --- | --- | --- |
| Journal Title | | |  |
| Year | | |  |
|  | | | |
| Article Characteristics | | |  |
|  | Drug | |  |
|  | Age of Experimental Drug | |  |
|  | Comparator | |  |
|  | Funding | |  |
|  | Study Location* | |  |
|  | Corresponding Author Location* | |  |
|  | Conclusion | |  |
|  | | | |
| COSTS | | |  |
|  | Actual Drug Cost | Reference† |  |
|  |  | No Reference† |  |
|  | Actual Health Care Cost | Reference |  |
|  |  | No Reference |  |
|  | Comparison of Drug Cost | Reference |  |
|  |  | No Reference |  |
|  | Comparison of Health Care Cost | Reference |  |
|  |  | No Reference |  |
|  | Any mention of drug costs | Reference |  |
|  |  | No Reference |  |
|  | Any mention of health care costs | Reference |  |
|  |  | No Reference |  |

* Location = Country

† Reference refers to the presence of a citation linked to any cost information.
