# Supplementary material for: Do Randomized Controlled Trials Discuss Healthcare Costs?
Source: PLoS One. 2010 Aug 23;5(8):e12318. doi: 10.1371/journal.pone.0012318 (PMC2925897; doi:10.1371/journal.pone.0012318)
Supplement: Appendix S2 — Articles Included (0.11 MB DOC) [file pone.0012318.s002.doc]

**Appendix S2: Articles Included**

**JAMA**

1. Albers GW, Diener HC, Frison L, Grind M, Nevinson M, *et al*; SPORTIF Executive Steering Committee for the SPORTIF V Investigators (2005) Ximelagatran vs warfarin for stroke prevention in patients with nonvalvular atrial fibrillation: a randomized trial. JAMA 293(6): 690-8.

2. Sato Y, Honda Y, Iwamoto J, Kanoko T, Satoh K (2005) Effect of folate and mecobalamin on hip fractures in patients with stroke: a randomized controlled trial. JAMA 293:1082-8.

3. Raitt MH, Connor WE, Morris C, Kron J, Halperin B, *et al* (2005) Fish oil supplementation and risk of ventricular tachycardia and ventricular fibrillation in patients with implantable defibrillators: a randomized controlled trial. JAMA 293:2884-91.

4. Cirillo DJ, Wallace RB, Rodabough RJ, Greenland P, LaCroix AZ *et al* (2005) Effect of estrogen therapy on gallbladder disease. JAMA 293:330-9.

5. Grant AD, Charalambous S, Fielding KL, Day JH, Corbett EL, *et al* (2005) Effect of routine isoniazid preventive therapy on tuberculosis incidence among HIV-infected men in South Africa: a novel randomized incremental recruitment study. JAMA 293:2719-25.

6. Yusuf S, Mehta SR, Xie C, Ahmed RJ, Xavier D, *et al*; CREATE Trial Group Investigators (2005) Effects of reviparin, a low-molecular-weight heparin, on mortality, reinfarction, and strokes in patients with acute myocardial infarction presenting with ST-segment elevation. JAMA 293:427-35.

7. Collins ED, Kleber HD, Whittington RA, Heitler NE (2005) Anesthesia-assisted vs buprenorphine- or clonidine-assisted heroin detoxification and naltrexone induction: a randomized trial. JAMA 294:903-13.

8. Pedersen TR, Faergeman O, Kastelein JJ, Olsson AG, Tikkanen MJ, *et al*; Incremental Decrease in End Points Through Aggressive Lipid Lowering (IDEAL) Study Group (2005) High-dose atorvastatin vs usual-dose simvastatin for secondary prevention after myocardial infarction: the IDEAL study: a randomized controlled trial. JAMA 294:2437-45.

9. Lee IM, Cook NR, Gaziano JM, Gordon D, Ridker PM, *et al* (2005) Vitamin E in the primary prevention of cardiovascular disease and cancer: the Women's Health Study: a randomized controlled trial. JAMA 294:56-65.

10. Pichichero ME, Rennels MB, Edwards KM, Blatter MM, Marshall GS, *et al* (2005) Combined tetanus, diphtheria, and 5-component pertussis vaccine for use in adolescents and adults. JAMA 293:3003-11.

11. Pediatric OCD Treatment Study (POTS) Team (2004) Cognitive-behavior therapy, sertraline, and their combination for children and adolescents with obsessive-compulsive disorder: the Pediatric OCD Treatment Study (POTS) randomized controlled trial. JAMA 292:1969-76.

12. Chaitman BR, Pepine CJ, Parker JO, Skopal J, Chumakova G, *et al*; Combination Assessment of Ranolazine In Stable Angina (CARISA) Investigators (2004) Effects of ranolazine with atenolol, amlodipine, or diltiazem on exercise tolerance and angina frequency in patients with severe chronic angina: a randomized controlled trial. JAMA 291:309-16.

13. Brandes JL, Saper JR, Diamond M, Couch JR, Lewis DW, *et al*; MIGR-002 Study Group (2004) Topiramate for migraine prevention: a randomized controlled trial. JAMA 291:965-73.

14. March J, Silva S, Petrycki S, Curry J, Wells K, et al; Treatment for Adolescents With Depression Study (TADS) Team (2004) Fluoxetine, cognitive-behavioral therapy, and their combination for adolescents with depression: Treatment for Adolescents With Depression Study (TADS) randomized controlled trial. JAMA 292:807-20.

15. Wiegman A, Hutten BA, de Groot E, Rodenburg J, Bakker HD, *et al* (2004) Efficacy and safety of statin therapy in children with familial hypercholesterolemia: a randomized controlled trial. JAMA 292:331-7.

16. Nissen SE, Tuzcu EM, Schoenhagen P, Brown BG, Ganz P, *et al*; REVERSAL Investigators (2004) Effect of intensive compared with moderate lipid-lowering therapy on progression of coronary atherosclerosis: a randomized controlled trial. JAMA 291:1071-80.

17. Cushman M, Kuller LH, Prentice R, Rodabough RJ, Psaty BM, *et al*; Women's Health Initiative Investigators (2004) Estrogen plus progestin and risk of venous thrombosis. JAMA 292:1573-80.

18. Blacker CV, Greenwood DT, Wesnes KA, Wilson R, Woodward C, *et al* (2004)

Effect of galantamine hydrobromide in chronic fatigue syndrome: a randomized controlled trial. JAMA 292:1195-204.

19. Saag MS, Cahn P, Raffi F, Wolff M, Pearce D, *et al*; FTC-301A Study Team (2004) Efficacy and safety of statin therapy in children with familial hypercholesterolemia: a randomized controlled trial. JAMA 292:331-7.

20. Gheorghiade M, Gattis WA, O'Connor CM, Adams KF Jr, Elkayam U, *et al*; Acute and Chronic Therapeutic Impact of a Vasopressin Antagonist in Congestive Heart Failure (ACTIV in CHF) Investigators (2004) Effects of tolvaptan, a vasopressin antagonist, in patients hospitalized with worsening heart failure: a randomized controlled trial. JAMA 291:1963-71.

21. Szapary PO, Wolfe ML, Bloedon LT, Cucchiara AJ, DerMarderosian AH, *et al* (2003) Guggulipid for the treatment of hypercholesterolemia: a randomized controlled trial. JAMA 290:765-72.

22. Saiman L, Marshall BC, Mayer-Hamblett N, Burns JL, Quittner AL, *et al*; Macrolide Study Group (2003) Azithromycin in patients with cystic fibrosis chronically infected with Pseudomonas aeruginosa: a randomized controlled trial. JAMA 290:1749-56.

23. Gorelick PB, Richardson D, Kelly M, Ruland S, Hung E, *et al*; African American Antiplatelet Stroke Prevention Study Investigators (2003). Aspirin and ticlopidine for prevention of recurrent stroke in black patients: a randomized trial. JAMA 289:2947-57.

24. Aisen PS, Schafer KA, Grundman M, Pfeiffer E, Sano M, *et al*; Alzheimer's Disease Cooperative Study (2003) Effects of rofecoxib or naproxen vs placebo on Alzheimer disease progression: a randomized controlled trial. JAMA 289:2819-26.

25. Tronvik E, Stovner LJ, Helde G, Sand T, Bovim G (2003) Prophylactic treatment of migraine with an angiotensin II receptor blocker: a randomized controlled trial. JAMA 289:65-9.

26. Gadde KM, Franciscy DM, Wagner HR 2nd, Krishnan KR (2003) Zonisamide for weight loss in obese adults: a randomized controlled trial. JAMA 289:1820-5.

27. Clyde PW, Harari AE, Getka EJ, Shakir KM (2003) Combined levothyroxine plus liothyronine compared with levothyroxine alone in primary hypothyroidism: a randomized controlled trial. JAMA 290:2952-8.

28. Buvanendran A, Kroin JS, Tuman KJ, Lubenow TR, Elmofty D, et al (2003) Effects of perioperative administration of a selective cyclooxygenase 2 inhibitor on pain management and recovery of function after knee replacement: a randomized controlled trial. JAMA 290:2411-8.

29. Stearns V, Beebe KL, Iyengar M, Dube E (2003) Paroxetine controlled release in the treatment of menopausal hot flashes: a randomized controlled trial. JAMA 289:2827-34.

30. Greenspan SL, Resnick NM, Parker RA (2003) Combination therapy with hormone replacement and alendronate for prevention of bone loss in elderly women: a randomized controlled trial. JAMA 289:2525-33.

31. Tariot PN, Farlow MR, Grossberg GT, Graham SM, McDonald S, et al; Memantine Study Group (2004) Memantine treatment in patients with moderate to severe Alzheimer disease already receiving donepezil: a randomized controlled trial. JAMA 291:317-24.

**N Engl J Med**

1. Groszmann RJ, Garcia-Tsao G, Bosch J, Grace ND, Burroughs AK, *et al*; Portal Hypertension Collaborative Group (2005) Beta-blockers to prevent gastroesophageal varices in patients with cirrhosis. N Engl J Med 353:2254-61.

2. Abraham E, Laterre PF, Garg R, Levy H, Talwar D, *et al*; Administration of Drotrecogin Alfa (Activated) in Early Stage Severe Sepsis (ADDRESS) Study Group (2005) Drotrecogin alfa (activated) for adults with severe sepsis and a low risk of death. N Engl J Med 353:1332-41.

3. Ridker PM, Cannon CP, Morrow D, Rifai N, Rose LM, *et al* (2005) C-reactive protein levels and outcomes after statin therapy. N Engl J Med 352:20-8.

4. Galie N, Ghofrani HA, Torbicki A, Barst RJ, Rubin LJ, *et al* (2005) Sildenafil citrate therapy for pulmonary arterial hypertension. N Engl J Med. 2005;353(20):2148-57.

5. Chan FK, Ching JY, Hung LC, Wong VW, Leung VK, et al (2005) Clopidogrel versus aspirin and esomeprazole to prevent recurrent ulcer bleeding. N Engl J Med 352:238-44.

6. Sabatine MS, Cannon CP, Gibson CM, Lopez-Sendon JL, Montalescot G, *et al* (2005) Addition of clopidogrel to aspirin and fibrinolytic therapy for myocardial infarction with ST-segment elevation. N Engl J Med 352:1179-89.

7. Singh BN, Singh SN, Reda DJ, Tang XC, Lopez B, *et al* (2005) Amiodarone versus sotalol for atrial fibrillation. N Engl J Med 352:1861-72.

8. LaRosa JC, Grundy SM, Waters DD, Shear C, Barter P, *et al* (2005) Intensive lipid lowering with atorvastatin in patients with stable coronary disease. N Engl J Med 352:1425-35.

9. Chimowitz MI, Lynn MJ, Howlett-Smith H, Stern BJ, Hertzberg VS, *et al* (2005) Comparison of warfarin and aspirin for symptomatic intracranial arterial stenosis. N Engl J Med 352:1305-16.

10. Lieberman JA, Stroup TS, McEvoy JP, Swartz MS, Rosenheck RA, *et al* (2005) Effectiveness of antipsychotic drugs in patients with chronic schizophrenia. N Engl J Med 353:1209-23.

11. Tannock IF, de Wit R, Berry WR, Horti J, Pluzanska A, *et al* (2004) Docetaxel plus prednisone or mitoxantrone plus prednisone for advanced prostate cancer. N Engl J Med 351:1502-12.

12. Bjornson CL, Klassen TP, Williamson J, Brant R, Mitton C, *et al* (2004) A randomized trial of a single dose of oral dexamethasone for mild croup. N Engl J Med 351:1306-13.

13. Strupp M, Zingler VC, Arbusow V, Niklas D, Maag KP, *et al* (2004) Methylprednisolone, valacyclovir, or the combination for vestibular neuritis. N Engl J Med 351:354-61.

14. Lallemant M, Jourdain G, Le Coeur S, Mary JY, Ngo-Giang-Huong N, *et al* (2004) Single-dose perinatal nevirapine plus standard zidovudine to prevent mother-to-child transmission of HIV-1 in Thailand. N Engl J Med 351:217-28.

15. Block GA, Martin KJ, de Francisco AL, Turner SA, Avram MM, *et al* (2004) Cinacalcet for secondary hyperparathyroidism in patients receiving hemodialysis. N Engl J Med 350:1516-25.

16. Corey L, Wald A, Patel R, Sacks SL, Tyring SK, *et al* (2004) Once-daily valacyclovir to reduce the risk of transmission of genital herpes. N Engl J Med 350:11-20.

17. Emre M, Aarsland D, Albanese A, Byrne EJ, Deuschl G, *et al* (2004) Rivastigmine for dementia associated with Parkinson's disease. N Engl J Med 351:2509-18.

18. Barnett AH, Bain SC, Bouter P, Karlberg B, Madsbad S, *et al* (2004). Angiotensin-receptor blockade versus converting-enzyme inhibition in type 2 diabetes and nephropathy. N Engl J Med 351:1952-61.

19. Thwaites GE, Nguyen DB, Nguyen HD, Hoang TQ, Do TT, *et al* (2004) Dexamethasone for the treatment of tuberculous meningitis in adolescents and adults. N Engl J Med 351:1741-51.

20. Sobel JD, Wiesenfeld HC, Martens M, Danna P, Hooton TM, *et al* (2004). Maintenance fluconazole therapy for recurrent vulvovaginal candidiasis. N Engl J Med 351:876-83.

21. Rowbotham MC, Twilling L, Davies PS, Reisner L, Taylor K, *et al* (2003) Oral opioid therapy for chronic peripheral and central neuropathic pain. N Engl J Med 348:1223-32.

22. McConnell JD, Roehrborn CG, Bautista OM, Andriole GL Jr, Dixon CM, *et al* (2003) The long-term effect of doxazosin, finasteride, and combination therapy on the clinical progression of benign prostatic hyperplasia. N Engl J Med 349:2387-98.

23. Finkelstein JS, Hayes A, Hunzelman JL, Wyland JJ, Lee H, *et al* (2003) The effects of parathyroid hormone, alendronate, or both in men with osteoporosis. N Engl J Med 349:1216-26.

24. Yanovski JA, Rose SR, Municchi G, Pescovitz OH, Hill SC, *et al* (2003) Treatment with a luteinizing hormone-releasing hormone agonist in adolescents with short stature. N Engl J Med 348:908-17.

25. Baron JA, Cole BF, Sandler RS, Haile RW, Ahnen D, *et al* (2003) A randomized trial of aspirin to prevent colorectal adenomas. N Engl J Med 348:891-9.

26. Reisberg B, Doody R, Stoffler A, Schmitt F, Ferris S, *et al* (2003) Memantine in moderate-to-severe Alzheimer's disease. N Engl J Med 348:1333-41.

27. Pfeffer MA, McMurray JJ, Velazquez EJ, Rouleau JL, Kober L, *et al* (2003) Valsartan, captopril, or both in myocardial infarction complicated by heart failure, left ventricular dysfunction, or both. N Engl J Med 349:1893-906.

28. Schulman S, Wahlander K, Lundstrom T, Clason SB, Eriksson H (2003) Secondary prevention of venous thromboembolism with the oral direct thrombin inhibitor ximelagatran. N Engl J Med 349:1713-21.

29. Black DM, Greenspan SL, Ensrud KE, Palermo L, McGowan JA, *et al* (2003) The effects of parathyroid hormone and alendronate alone or in combination in postmenopausal osteoporosis. N Engl J Med 349:1207-15.

30. Fudala PJ, Bridge TP, Herbert S, Williford WO, Chiang CN, *et al* (2003) Office-based treatment of opiate addiction with a sublingual-tablet formulation of buprenorphine and naloxone. N Engl J Med 349:949-58.

31. Leonardi CL, Powers JL, Matheson RT, Goffe BS, Zitnik R, *et al* (2003) Etanercept as monotherapy in patients with psoriasis. N Engl J Med 349:2014-22.

32. Taylor AL, Ziesche S, Yancy C, Carson P, D'Agostino R Jr, et al (2004) Combination of isosorbide dinitrate and hydralazine in blacks with heart failure. N Engl J Med 351:2049-57.

**BMJ**

1. Chandramohan D, Owusu-Agyei S, Carneiro I, Awine T, Amponsa-Achiano K, *et al* (2005) Cluster randomised trial of intermittent preventive treatment for malaria in infants in area of high, seasonal transmission in Ghana. BMJ 331:727-33.

2. Corwin P, Toop L, McGeoch G, Than M, Wynn-Thomas S, *et al* (2005) Randomised controlled trial of intravenous antibiotic treatment for cellulitis at home compared with hospital. BMJ 330:129.

3. Stutchfield P, Whitaker R, Russell I (2005) Antenatal betamethasone and incidence of neonatal respiratory distress after elective caesarean section: pragmatic randomised trial. BMJ 331:662.

4. Szegedi A, Kohnen R, Dienel A, Kieser M (2005) Acute treatment of moderate to severe depression with hypericum extract WS 5570 (St John's wort): randomised controlled double blind non-inferiority trial versus paroxetine. BMJ 330:503.

5. Avenell A, Campbell MK, Cook JA, Hannaford PC, Kilonzo MM, *et al* (2005) Effect of multivitamin and multimineral supplements on morbidity from infections in older people (MAVIS trial): pragmatic, randomised, double blind, placebo controlled trial. BMJ 331:324-9.

6. Benn CS, Martins C, Rodrigues A, Jensen H, Lisse IM, *et al* (2005) Randomised study of effect of different doses of vitamin A on childhood morbidity and mortality. BMJ 331:1428-32.

7. Hoj L, Cardoso P, Nielsen BB, Hvidman L, Nielsen J, *et al* (2005) Effect of sublingual misoprostol on severe postpartum haemorrhage in a primary health centre in Guinea-Bissau: randomised double blind clinical trial. BMJ 331:723.

8. Ballard C, Margallo-Lana M, Juszczak E, Douglas S, Swann A, *et al* (2005) Quetiapine and rivastigmine and cognitive decline in Alzheimer's disease: randomised double blind placebo controlled trial. BMJ 330:874.

9. Richards D, Toop L, Chambers S, Fletcher L (2005) Response to antibiotics of women with symptoms of urinary tract infection but negative dipstick urine test results: double blind randomised controlled trial. BMJ 331:143.

10. Bruno S, Maisonneuve P, Castellana P, Rotmensz N, Rossi S, *et al* (2005) Incidence and risk factors for non-alcoholic steatohepatitis: prospective study of 5408 women enrolled in Italian tamoxifen chemoprevention trial. BMJ 330:932.

11. Marre M, Lievre M, Chatellier G, Mann JF, Passa P, *et al* (2004) Effects of low dose ramipril on cardiovascular and renal outcomes in patients with type 2 diabetes and raised excretion of urinary albumin: randomised, double blind, placebo controlled trial (the DIABHYCAR study). BMJ 328:495.

12. Agarwal G, Awasthi S, Kabra SK, Kaul A, Singhi S, *et al* (2004) Three day versus five day treatment with amoxicillin for non-severe pneumonia in young children: a multicentre randomised controlled trial. BMJ 328:791.

13. Pirotta M, Gunn J, Chondros P, Grover S, O'Malley P, *et al* (2004) Effect of lactobacillus in preventing post-antibiotic vulvovaginal candidiasis: a randomised controlled trial. BMJ 329:548.

14. Svendsen KB, Jensen TS, Bach FW (2004) Does the cannabinoid dronabinol reduce central pain in multiple sclerosis? Randomised double blind placebo controlled crossover trial. BMJ 329:253.

15. Smith WC, Anderson AM, Withington SG, van Brakel WH, Croft RP, *et al* (2004) Steroid prophylaxis for prevention of nerve function impairment in leprosy: randomised placebo controlled trial (TRIPOD 1). BMJ 328:1459.

16. Gertsch JH, Basnyat B, Johnson EW, Onopa J, Holck PS (2004) Randomised, double blind, placebo controlled comparison of ginkgo biloba and acetazolamide for prevention of acute mountain sickness among Himalayan trekkers: the prevention of high altitude illness trial (PHAIT). BMJ 328:797.

17. Miranda-Filho Dde B, Ximenes RA, Barone AA, Vaz VL, Vieira AG, *et al* (2004) Randomised controlled trial of tetanus treatment with antitetanus immunoglobulin by the intrathecal or intramuscular route. BMJ 328:615.

18. Koivunen P, Uhari M, Luotonen J, Kristo A, Raski R, *et al* (2004) Adenoidectomy versus chemoprophylaxis and placebo for recurrent acute otitis media in children aged under 2 years: randomised controlled trial. BMJ 328:487.

19. Bayram N, van Wely M, Kaaijk EM, Bossuyt PM, van der Veen F (2004) Using an electrocautery strategy or recombinant follicle stimulating hormone to induce ovulation in polycystic ovary syndrome: randomised controlled trial. BMJ 328:192.

20. Vas J, Mendez C, Perea-Milla E, Vega E, Panadero MD, *et al* (2004) Acupuncture as a complementary therapy to the pharmacological treatment of osteoarthritis of the knee: randomised controlled trial. BMJ 329:1216.

21. Zwart S, Rovers MM, de Melker RA, Hoes AW (2003) Penicillin for acute sore throat in children: randomised, double blind trial. BMJ 327:1324.

22. Bjermer L, Bisgaard H, Bousquet J, Fabbri LM, Greening AP, *et al* (2003) Montelukast and fluticasone compared with salmeterol and fluticasone in protecting against asthma exacerbation in adults: one year, double blind, randomised, comparative trial. BMJ 327:891.

23. Berth-Jones J, Damstra RJ, Golsch S, Livden JK, Van Hooteghem O, *et al* (2003) Twice weekly fluticasone propionate added to emollient maintenance treatment to reduce risk of relapse in atopic dermatitis: randomised, double blind, parallel group study. BMJ 326:1367.

24. Verdon F, Burnand B, Stubi CL, Bonard C, Graff M, *et al* (2003) Iron supplementation for unexplained fatigue in non-anaemic women: double blind randomised placebo controlled trial. BMJ 326:1124.

25. Trivedi DP, Doll R, Khaw KT (2003) Effect of four monthly oral vitamin D3 (cholecalciferol) supplementation on fractures and mortality in men and women living in the community: randomised double blind controlled trial. BMJ 326:469.

26. Rahmathullah L, Tielsch JM, Thulasiraj RD, Katz J, Coles C, *et al* (2003) Impact of supplementing newborn infants with vitamin A on early infant mortality: community based randomised trial in southern India. BMJ 327:254.

27. van Balen FA, Smit WM, Zuithoff NP, Verheij TJ (2003) Clinical efficacy of three common treatments in acute otitis externa in primary care: randomised controlled trial. BMJ 327:1201-5.

28. Schlagenhauf P, Tschopp A, Johnson R, Nothdurft HD, Beck B, *et al* (2003) Tolerability of malaria chemoprophylaxis in non-immune travellers to sub-Saharan Africa: multicentre, randomised, double blind, four arm study. BMJ 327:1078.

29. Abernethy AP, Currow DC, Frith P, Fazekas BS, McHugh A, *et al* (2003) Randomised, double blind, placebo controlled crossover trial of sustained release morphine for the management of refractory dyspnoea. BMJ 327:523-8.

30. Radcliffe MJ, Lewith GT, Turner RG, Prescott P, Church MK, *et al* (2003) Enzyme potentiated desensitisation in treatment of seasonal allergic rhinitis: double blind randomised controlled study. BMJ 327:251-4.

**Lancet**

1. Cutts FT, Zaman SM, Enwere G, Jaffar S, Levine OS, *et al* (2005) Efficacy of nine-valent pneumococcal conjugate vaccine against pneumonia and invasive pneumococcal disease in The Gambia: randomised, double-blind, placebo-controlled trial. Lancet 365:1139-46.

2. Rose PW, Harnden A, Brueggemann AB, Perera R, Sheikh A, *et al* (2005) Chloramphenicol treatment for acute infective conjunctivitis in children in primary care: a randomised double-blind placebo-controlled trial. Lancet 366:37-43.

3. Rabe KF, Bateman ED, O'Donnell D, Witte S, Bredenbroker D, *et al* (2005) Roflumilast--an oral anti-inflammatory treatment for chronic obstructive pulmonary disease: a randomised controlled trial. Lancet 366:563-71.

4. Thatcher N, Chang A, Parikh P, Rodrigues Pereira J, Ciuleanu T, et al (2005) Gefitinib plus best supportive care in previously treated patients with refractory advanced non-small-cell lung cancer: results from a randomised, placebo-controlled, multicentre study (Iressa Survival Evaluation in Lung Cancer). Lancet 366:1527-37.

5. Chen ZM, Pan HC, Chen YP, Peto R, Collins R, *et al* (2005) Early intravenous then oral metoprolol in 45,852 patients with acute myocardial infarction: randomised placebo-controlled trial. Lancet 366:1622-32.

6. Ruggenenti P, Perna A, Loriga G, Ganeva M, Ene-Iordache B, *et al* (2005) Blood-pressure control for renoprotection in patients with non-diabetic chronic renal disease (REIN-2): multicentre, randomised controlled trial. Lancet 365:939-46.

7. Granger BB, Swedberg K, Ekman I, Granger CB, Olofsson B, *et al* (2005) Adherence to candesartan and placebo and outcomes in chronic heart failure in the CHARM programme: double-blind, randomised, controlled clinical trial. Lancet 366:2005-11.

8. Dondorp A, Nosten F, Stepniewska K, Day N, White N (2005) Artesunate versus quinine for treatment of severe falciparum malaria: a randomised trial. Lancet 366:717-25.

9. Zhang S, Wang S, Li Q, Yao S, Zeng B, *et al* (2005) Capillary leak syndrome in children with C4A-deficiency undergoing cardiac surgery with cardiopulmonary bypass: a double-blind, randomised controlled study. Lancet 366:556-62.

10. Van Gaal LF, Rissanen AM, Scheen AJ, Ziegler O, Rossner S (2005) Effects of the cannabinoid-1 receptor blocker rimonabant on weight reduction and cardiovascular risk factors in overweight patients: 1-year experience from the RIO-Europe study. Lancet 365:1389-97.

11. Klareskog L, van der Heijde D, de Jager JP, Gough A, Kalden J, *et al* (2004) Therapeutic effect of the combination of etanercept and methotrexate compared with each treatment alone in patients with rheumatoid arthritis: double-blind randomised controlled trial. Lancet 363:675-81.

12. Muir KW, Lees KR, Ford I, Davis S (2004) Magnesium for acute stroke (Intravenous Magnesium Efficacy in Stroke trial): randomised controlled trial. Lancet 363:439-45.

13. Tran TH, Dolecek C, Pham PM, Nguyen TD, Nguyen TT, *et al* (2004) Dihydroartemisinin-piperaquine against multidrug-resistant Plasmodium falciparum malaria in Vietnam: randomised clinical trial.

Lancet 363:18-22.

14. Ozolins M, Eady EA, Avery AJ, Cunliffe WJ, Po AL, *et al* (2004) Comparison of five antimicrobial regimens for treatment of mild to moderate inflammatory facial acne vulgaris in the community: randomised controlled trial.

Lancet 364:2188-95.

15. Van Overmeire B, Allegaert K, Casaer A, Debauche C, Decaluwe W, *et al* (2004) Prophylactic ibuprofen in premature infants: a multicentre, randomised, double-blind, placebo-controlled trial. Lancet 364:1945-9.

16. Diener HC, Bogousslavsky J, Brass LM, Cimminiello C, Csiba L, *et al* (2004) Aspirin and clopidogrel compared with clopidogrel alone after recent ischaemic stroke or transient ischaemic attack in high-risk patients (MATCH): randomised, double-blind, placebo-controlled trial. Lancet 364:331-7.

17. Brooks WA, Yunus M, Santosham M, Wahed MA, Nahar K, *et al* (2004) Zinc for severe pneumonia in very young children: double-blind placebo-controlled trial.

Lancet 363:1683-8.

18. Courtney C, Farrell D, Gray R, Hills R, Lynch L, *et al* (2004) Long-term donepezil treatment in 565 patients with Alzheimer's disease (AD2000): randomised double-blind trial. Lancet 363:2105-15.

19. McCarey DW, McInnes IB, Madhok R, Hampson R, Scherbakov O, *et al* (2004) Trial of Atorvastatin in Rheumatoid Arthritis (TARA): double-blind, randomised placebo-controlled trial. Lancet 363:2015-21.

20. Alloueche A, Bailey W, Barton S, Bwika J, Chimpeni P, *et al* (2004) Comparison of chlorproguanil-dapsone with sulfadoxine-pyrimethamine for the treatment of uncomplicated falciparum malaria in young African children: double-blind randomised controlled trial. Lancet 363:1843-8.

21. Oommen A, Lambert PC, Grigg J (2003) Efficacy of a short course of parent-initiated oral prednisolone for viral wheeze in children aged 1-5 years: randomised controlled trial. Lancet 362:1433-8.

22. Wallentin L, Wilcox RG, Weaver WD, Emanuelsson H, Goodvin A, *et al* (2003) Oral ximelagatran for secondary prophylaxis after myocardial infarction: the ESTEEM randomised controlled trial. Lancet 362:789-97.

23. Hughes R, Goldkorn A, Masoli M, Weatherall M, Burgess C, *et al* (2003) Use of isotonic nebulised magnesium sulphate as an adjuvant to salbutamol in treatment of severe asthma in adults: randomised placebo-controlled trial. Lancet 361:2114-7.

24. Holdaas H, Fellstrom B, Jardine AG, Holme I, Nyberg G, *et al* (2003) Effect of fluvastatin on cardiac outcomes in renal transplant recipients: a multicentre, randomised, placebo-controlled trial. Lancet 361:2024-31.

25. Johnson BA, Ait-Daoud N, Bowden CL, DiClemente CC, Roache JD, *et al* (2003) Oral topiramate for treatment of alcohol dependence: a randomised controlled trial. Lancet 361:1677-85.

26. Cercek B, Shah PK, Noc M, Zahger D, Zeymer U, *et al* (2003) Effect of short-term treatment with azithromycin on recurrent ischaemic events in patients with acute coronary syndrome in the Azithromycin in Acute Coronary Syndrome (AZACS) trial: a randomised controlled trial. Lancet 361:809-13.

27. Pauwels RA, Pedersen S, Busse WW, Tan WC, Chen YZ, *et al* (2003) Early intervention with budesonide in mild persistent asthma: a randomised, double-blind trial. Lancet 361:1071-6.

28. Calverley P, Pauwels R, Vestbo J, Jones P, Pride N, *et al* (2003) Combined salmeterol and fluticasone in the treatment of chronic obstructive pulmonary disease: a randomised controlled trial. Lancet 361:449-56.

29. Collins R, Armitage J, Parish S, Sleigh P, Peto R (2003) MRC/BHF Heart Protection Study of cholesterol-lowering with simvastatin in 5963 people with diabetes: a randomised placebo-controlled trial. Lancet 361:2005-16.

30. Zajicek J, Fox P, Sanders H, Wright D, Vickery J, *et al* (2003) Cannabinoids for treatment of spasticity and other symptoms related to multiple sclerosis (CAMS study): multicentre randomised placebo-controlled trial. Lancet 362:1517-26.

31. Oommen A, Lambert PC, Grigg J (2003) Efficacy of a short course of parent-initiated oral prednisolone for viral wheeze in children aged 1-5 years: randomised controlled trial. Lancet 362:1433-8.

32. Dahlof B, Sever PS, Poulter NR, Wedel H, Beevers DG, *et al* (2005) Prevention of cardiovascular events with an antihypertensive regimen of amlodipine adding perindopril as required versus atenolol adding bendroflumethiazide as required, in the Anglo-Scandinavian Cardiac Outcomes Trial-Blood Pressure Lowering Arm (ASCOT-BPLA): a multicentre randomised controlled trial. Lancet 366:895-906.

**Annals of Internal Medicine**

1. Buyon JP, Petri MA, Kim MY, Kalunian KC, Grossman J, *et al* (2005) The effect of combined estrogen and progesterone hormone replacement therapy on disease activity in systemic lupus erythematosus: a randomized trial. Ann Intern Med 142:953-62.

2. Niewoehner DE, Rice K, Cote C, Paulson D, Cooper JA Jr, *et al* (2005) Prevention of exacerbations of chronic obstructive pulmonary disease with tiotropium, a once-daily inhaled anticholinergic bronchodilator: a randomized trial. Ann Intern Med 143:317-26.

3. Wong SM, Hui AC, Tong PY, Poon DW, Yu E, *et al* (2005) Treatment of lateral epicondylitis with botulinum toxin: a randomized, double-blind, placebo-controlled trial. Ann Intern Med 143:793-7.

4. Chan HL, Leung NW, Hui AY, Wong VW, Liew CT, *et al* (2005) A randomized, controlled trial of combination therapy for chronic hepatitis B: comparing pegylated interferon-alpha2b and lamivudine with lamivudine alone. Ann Intern Med 142:240-50.

5. He J, Gu D, Wu X, Chen J, Duan X, *et al* (2005) Effect of soybean protein on blood pressure: a randomized, controlled trial. Ann Intern Med 143:1-9.

6. DuPont HL, Jiang ZD, Okhuysen PC, Ericsson CD, de la Cabada FJ, *et al* (2005) A randomized, double-blind, placebo-controlled trial of rifaximin to prevent travelers' diarrhea. Ann Intern Med 142:805-12.

7. Clifford DB, Evans S, Yang Y, Acosta EP, Goodkin K, *et al* (2005) Impact of efavirenz on neuropsychological performance and symptoms in HIV-infected individuals. Ann Intern Med 143:714-21.

8. Escobar-Morreale HF, Botella-Carretero JI, Gomez-Bueno M, Galan JM, Barrios V, *et al* (2005) Thyroid hormone replacement therapy in primary hypothyroidism: a randomized trial comparing L-thyroxine plus liothyronine with L-thyroxine alone. Ann Intern Med 142:412-24.

9. Orchard TJ, Temprosa M, Goldberg R, Haffner S, Ratner R, *et al* (2005) The effect of metformin and intensive lifestyle intervention on the metabolic syndrome: the Diabetes Prevention Program randomized trial. Ann Intern Med 142:611-9.

10. Whitney EJ, Krasuski RA, Personius BE, Michalek JE, Maranian AM, *et al* (2005) A randomized trial of a strategy for increasing high-density lipoprotein cholesterol levels: effects on progression of coronary heart disease and clinical events. Ann Intern Med 142:95-104.

11. Caporali R, Cimmino MA, Ferraccioli G, Gerli R, Klersy C, *et al* (2004) Prednisone plus methotrexate for polymyalgia rheumatica: a randomized, double-blind, placebo-controlled trial. Ann Intern Med 141:493-500.

12. Alexander RB, Propert KJ, Schaeffer AJ, Landis JR, Nickel JC, *et al* (2004) Ciprofloxacin or tamsulosin in men with chronic prostatitis/chronic pelvic pain syndrome: a randomized, double-blind trial. Ann Intern Med 141:581-9.

13. Ghofrani HA, Reichenberger F, Kohstall MG, Mrosek EH, Seeger T, *et al* (2004) Sildenafil increased exercise capacity during hypoxia at low altitudes and at Mount Everest base camp: a randomized, double-blind, placebo-controlled crossover trial. Ann Intern Med 141:169-77.

14. Buller HR, Davidson BL, Decousus H, Gallus A, Gent M, *et al* (2004) Fondaparinux or enoxaparin for the initial treatment of symptomatic deep venous thrombosis: a randomized trial. Ann Intern Med 140:867-73.

15. Hadigan C, Yawetz S, Thomas A, Havers F, Sax PE, *et al* (2004) Metabolic effects of rosiglitazone in HIV lipodystrophy: a randomized, controlled trial. Ann Intern Med 140:786-94.

16. Wertheim HF, Vos MC, Ott A, Voss A, Kluytmans JA, *et al* (2004) Mupirocin prophylaxis against nosocomial Staphylococcus aureus infections in nonsurgical patients: a randomized study. Ann Intern Med 140:419-25.

17. Hadziyannis SJ, Sette H Jr, Morgan TR, Balan V, Diago M, *et al* (2004) Peginterferon-alpha2a and ribavirin combination therapy in chronic hepatitis C: a randomized study of treatment duration and ribavirin dose. Ann Intern Med 140:346-55.

18. Bach RG, Cannon CP, Weintraub WS, DiBattiste PM, Demopoulos LA, *et al* (2004) The effect of routine, early invasive management on outcome for elderly patients with non-ST-segment elevation acute coronary syndromes. Ann Intern Med 141:186-95.

19. Donta ST, Engel CC Jr, Collins JF, Baseman JB, Dever LL, *et al* (2004) Benefits and harms of doxycycline treatment for Gulf War veterans' illnesses: a randomized, double-blind, placebo-controlled trial. Ann Intern Med 141:85-94.

20. Yuan CS, Wei G, Dey L, Karrison T, Nahlik L, *et al* (2004) Brief communication: American ginseng reduces warfarin's effect in healthy patients: a randomized, controlled Trial. Ann Intern Med 141:23-7.

21. Lisse JR, Perlman M, Johansson G, Shoemaker JR, Schechtman J, *et al* (2003). Gastrointestinal tolerability and effectiveness of rofecoxib versus naproxen in the treatment of osteoarthritis: a randomized, controlled trial. Ann Intern Med 139:539-46.

22. Abrams DI, Hilton JF, Leiser RJ, Shade SB, Elbeik TA, *et al* (2003) Short-term effects of cannabinoids in patients with HIV-1 infection: a randomized, placebo-controlled clinical trial. Ann Intern Med 139:258-66.

23. Squires K, Pozniak AL, Pierone G Jr, Steinhart CR, Berger D, *et al* (2003) Tenofovir disoproxil fumarate in nucleoside-resistant HIV-1 infection: a randomized trial. Ann Intern Med 139:313-20.

24, Devereux RB, Dahlof B, Kjeldsen SE, Julius S, Aurup P, *et al* (2003) Effects of losartan or atenolol in hypertensive patients without clinically evident vascular disease: a substudy of the LIFE randomized trial. Ann Intern Med 139:169-77.

25, Tonelli M, Moye L, Sacks FM, Kiberd B, Curhan G (2003) Pravastatin for secondary prevention of cardiovascular events in persons with mild chronic renal insufficiency. Ann Intern Med 138:98-104.

26. Kanaya AM, Herrington D, Vittinghoff E, Lin F, Grady D, *et al* (2003). Glycemic effects of postmenopausal hormone therapy: the Heart and Estrogen/progestin Replacement Study. A randomized, double-blind, placebo-controlled trial. Ann Intern Med 138:1-9.

27. Berl T, Hunsicker LG, Lewis JB, Pfeffer MA, Porush JG, *et al* (2003) Collaborative Study Group. Cardiovascular outcomes in the Irbesartan Diabetic Nephropathy Trial of patients with type 2 diabetes and overt nephropathy. Ann Intern Med 138:542-9.

28. Wormser GP, Ramanathan R, Nowakowski J, McKenna D, Holmgren D, *et al* (2003) Duration of antibiotic therapy for early Lyme disease. A randomized, double-blind, placebo-controlled trial. Ann Intern Med 138:697-704.

29. Zandbergen AA, Baggen MG, Lamberts SW, Bootsma AH, de Zeeuw D, *et al* (2003) Effect of losartan on microalbuminuria in normotensive patients with type 2 diabetes mellitus. A randomized clinical trial. Ann Intern Med 139:90-6.

30. Kovacs MJ, Rodger M, Anderson DR, Morrow B, Kells G, *et al* (2003) Comparison of 10-mg and 5-mg warfarin initiation nomograms together with low-molecular-weight heparin for outpatient treatment of acute venous thromboembolism. A randomized, double-blind, controlled trial. Ann Intern Med 138:714-9.

31. Rasaratnam B, Kaye D, Jennings G, Dudley F, Chin-Dusting J (2003) The effect of selective intestinal decontamination on the hyperdynamic circulatory state in cirrhosis. A randomized trial. Ann Intern Med 139:186-93

**Archives of Internal Medicine**

1. Eussen SJ, de Groot LC, Clarke R, Schneede J, Ueland PM, *et al* (2005) Oral cyanocobalamin supplementation in older people with vitamin B12 deficiency: a dose-finding trial. Arch Intern Med 165:1167-72.

2. Sato Y, Kanoko T, Satoh K, Iwamoto J (2005) The prevention of hip fracture with risedronate and ergocalciferol plus calcium supplementation in elderly women with Alzheimer disease: a randomized controlled trial. Arch Intern Med 165:1737-42.

3. Braunstein GD, Sundwall DA, Katz M, Shifren JL, Buster JE, *et al* (2005) Safety and efficacy of a testosterone patch for the treatment of hypoactive sexual desire disorder in surgically menopausal women: a randomized, placebo-controlled trial. Arch Intern Med 165:1582-9.

4. Durga J, van Tits LJ, Schouten EG, Kok FJ, Verhoef P (2005) Effect of lowering of homocysteine levels on inflammatory markers: a randomized controlled trial. Arch Intern Med 165:1388-94.

5. McClung MR, San Martin J, Miller PD, Civitelli R, Bandeira F, *et al* (2005) Opposite bone remodeling effects of teriparatide and alendronate in increasing bone mass. Arch Intern Med 165:1762-8.

6. Imazio M, Bobbio M, Cecchi E, Demarie D, Pomari F, *et al* (2005) Colchicine as first-choice therapy for recurrent pericarditis: results of the CORE (COlchicine for REcurrent pericarditis) trial. Arch Intern Med 165:1987-91.

7. Roos A, Linn-Rasker SP, van Domburg RT, Tijssen JP, Berghout A (2005) The starting dose of levothyroxine in primary hypothyroidism treatment: a prospective, randomized, double-blind trial. Arch Intern Med 165:1714-20.

8. Chow T, Browne V, Heileson HL, Wallace D, Anholm J, *et al* (2005) Ginkgo biloba and acetazolamide prophylaxis for acute mountain sickness: a randomized, placebo-controlled trial. Arch Intern Med 165:296-301.

9. Mulligan K, Zackin R, Clark RA, Alston-Smith B, Liu T, *et al* (2005) Effect of nandrolone decanoate therapy on weight and lean body mass in HIV-infected women with weight loss: a randomized, double-blind, placebo-controlled, multicenter trial. Arch Intern Med 165:578-85.

10. Tenenbaum A, Motro M, Fisman EZ, Tanne D, Boyko V, *et al* (2005) Bezafibrate for the secondary prevention of myocardial infarction in patients with metabolic syndrome. Arch Intern Med 165:1154-60.

11. Hepburn MJ, Dooley DP, Skidmore PJ, Ellis MW, Starnes WF, *et al* (2004) Comparison of short-course (5 days) and standard (10 days) treatment for uncomplicated cellulitis. Arch Intern Med 164:1669-74.

12. Jakkula M, Boucher TA, Beyendorff U, Conn SM, Johnson JE, *et al* (2004) A randomized trial of Chinese herbal medicines for the treatment of symptomatic hepatitis C. Arch Intern Med 164:1341-6.

13. Wiese J, McPherson S, Odden MC, Shlipak MG (2004) Effect of Opuntia ficus indica on symptoms of the alcohol hangover. Arch Intern Med 164:1334-40.

14. Yale SH, Liu K (2004) Echinacea purpurea therapy for the treatment of the common cold: a randomized, double-blind, placebo-controlled clinical trial. Arch Intern Med 164:1237-41.

15. Insull W Jr, McGovern ME, Schrott H, Thompson P, Crouse JR, *et al* (2004) Efficacy of extended-release niacin with lovastatin for hypercholesterolemia: assessing all reasonable doses with innovative surface graph analysis. Arch Intern Med 164:1121-7.

16. Prandoni P, Carnovali M, Marchiori A (2004) Subcutaneous adjusted-dose unfractionated heparin vs fixed-dose low-molecular-weight heparin in the initial treatment of venous thromboembolism. Arch Intern Med 164:1077-83.

17. Dolan S, Wilkie S, Aliabadi N, Sullivan MP, Basgoz N, *et al* (2004) Effects of testosterone administration in human immunodeficiency virus-infected women with low weight: a randomized placebo-controlled study. Arch Intern Med 164:897-904.

18. Reid IR, Eastell R, Fogelman I, Adachi JD, Rosen A *et al* (2004) A comparison of the effects of raloxifene and conjugated equine estrogen on bone and lipids in healthy postmenopausal women. Arch Intern Med 164:871-9.

19. Webster LJ, Michelakis ED, Davis T, Archer SL (2004) Use of sildenafil for safe improvement of erectile function and quality of life in men with New York Heart Association classes II and III congestive heart failure: a prospective, placebo-controlled, double-blind crossover trial. Arch Intern Med 164:514-20.

20. Hercberg S, Galan P, Preziosi P, Bertrais S, Mennen L, *et al* (2004) The SU.VI.MAX Study: a randomized, placebo-controlled trial of the health effects of antioxidant vitamins and minerals. Arch Intern Med 164:2335-42.

21. Superficial Thrombophlebitis Treated By Enoxaparin Study Group (2003) A pilot randomized double-blind comparison of a low-molecular-weight heparin, a nonsteroidal anti-inflammatory agent, and placebo in the treatment of superficial vein thrombosis. Arch Intern Med 163:1657-63.

22. Scroggie DA, Albright A, Harris MD (2003) The effect of glucosamine-chondroitin supplementation on glycosylated hemoglobin levels in patients with type 2 diabetes mellitus: a placebo-controlled, double-blinded, randomized clinical trial. Arch Intern Med 163:1587-90.

23. Eriksson BI, Lassen MR (2003) Duration of prophylaxis against venous thromboembolism with fondaparinux after hip fracture surgery: a multicenter, randomized, placebo-controlled, double-blind study. Arch Intern Med 163:1337-42.

24. Tzourio C, Anderson C, Chapman N, Woodward M, Neal B, *et al* (2003) Effects of blood pressure lowering with perindopril and indapamide therapy on dementia and cognitive decline in patients with cerebrovascular disease. Arch Intern Med 163:1069-75.

25. Ascott-Evans BH, Guanabens N, Kivinen S, Stuckey BG, Magaril CH, *et al* (2003) Alendronate prevents loss of bone density associated with discontinuation of hormone replacement therapy: a randomized controlled trial. Arch Intern Med 163:789-94.

26. Percheron G, Hogrel JY, Denot-Ledunois S, Fayet G, Forette F, *et al* (2003) Effect of 1-year oral administration of dehydroepiandrosterone to 60- to 80-year-old individuals on muscle function and cross-sectional area: a double-blind placebo-controlled trial. Arch Intern Med 163:720-7.

27. Case JP, Baliunas AJ, Block JA (2003) Lack of efficacy of acetaminophen in treating symptomatic knee osteoarthritis: a randomized, double-blind, placebo-controlled comparison trial with diclofenac sodium. Arch Intern Med 163:169-78.

28. Weiss SR, Cheng SL, Kourides IA, Gelfand RA, Landschulz WH (2003) Inhaled insulin provides improved glycemic control in patients with type 2 diabetes mellitus inadequately controlled with oral agents: a randomized controlled trial. Arch Intern Med 163:2277-82.

29. Bucher HC, Tschudi P, Young J, Periat P, Welge-Luussen A, *et al* (2003) Effect of amoxicillin-clavulanate in clinically diagnosed acute rhinosinusitis: a placebo-controlled, double-blind, randomized trial in general practice. Arch Intern Med 163:1793-8.

30. Hollenberg NK, Williams GH, Anderson R, Akhras KS, Bittman RM, *et al* (2003) Symptoms and the distress they cause: comparison of an aldosterone antagonist and a calcium channel blocking agent in patients with systolic hypertension. Arch Intern Med 163:1543-8.

31. Wagena EJ, Knipschild PG, Huibers MJ, Wouters EF, van Schayck CP (2005) Efficacy of bupropion and nortriptyline for smoking cessation among people at risk for or with chronic obstructive pulmonary disease. Arch Intern Med 165:2286-92.

32. Prochazka AV, Kick S, Steinbrunn C, Miyoshi T, Fryer GE (2004) A randomized trial of nortriptyline combined with transdermal nicotine for smoking cessation. Arch Intern Med 164:2229-33.
